# Large unexplained suite of chemically reactive compounds present in ambient air due to biomass fires

**DOI:** 10.1038/s41598-017-19139-3

**Published:** 2018-01-12

**Authors:** V. Kumar, B. P. Chandra, V. Sinha

**Affiliations:** Department of Earth and Environmental Sciences, Indian Institute of Science Education and Research Mohali, Sector 81, S. A. S. Nagar, Manauli PO, Punjab 140306 India

## Abstract

Biomass fires impact global atmospheric chemistry. The reactive compounds emitted and formed due to biomass fires drive ozone and organic aerosol formation, affecting both air quality and climate. Direct hydroxyl (OH) Reactivity measurements quantify total gaseous reactive pollutant loadings and comparison with measured compounds yields the fraction of unmeasured compounds. Here, we quantified the magnitude and composition of total OH reactivity in the north-west Indo-Gangetic Plain. More than 120% increase occurred in total OH reactivity (28 s^−1^ to 64 s^−1^) and from no missing OH reactivity in the normal summertime air, the missing OH reactivity fraction increased to ~40 % in the post-harvest summertime period influenced by large scale biomass fires highlighting presence of unmeasured compounds. Increased missing OH reactivity between the two summertime periods was associated with increased concentrations of compounds with strong photochemical source such as acetaldehyde, acetone, hydroxyacetone, nitromethane, amides, isocyanic acid and primary emissions of acetonitrile and aromatic compounds. Currently even the most detailed state-of-the art atmospheric chemistry models exclude formamide, acetamide, nitromethane and isocyanic acid and their highly reactive precursor alkylamines (e.g. methylamine, ethylamine, dimethylamine, trimethylamine). For improved understanding of atmospheric chemistry-air quality-climate feedbacks in biomass-fire impacted atmospheric environments, future studies should include these compounds.

## Introduction

Biomass burning is a widespread practice globally^1^, which causes soil nutrient loss and impacts atmospheric chemistry, air quality and climate^2–4^. Among all forms of biomass burning, agricultural waste burning is completely anthropogenic and its adverse environmental effects are exacerbated due to the short time period (few weeks) and large area, over which the activity occurs^1^. Of more than 3700 Tg of agricultural residue produced every year globally^5^, 10–25% is burnt openly^1^, injecting several hundred million metric tonnes of reactive pollutants into the atmosphere^3,6,7^. The north-western Indo-Gangetic Plain (N.W. IGP) is a critical demographic region of the world that serves as the agricultural “bread basket” of South Asia. Due to increasing anthropogenic activities, there is concern that air pollution in the form of increased surface ozone is adversely affecting crop yields^8^. A multi-year study has recently documented frequent exceedances in the 8h average national ambient air quality standard (NAAQS) for ozone^9^ (>60 % annually). In particular, the summertime air influenced by emissions from the agricultural biomass fires^9^ is characterized by the highest ambient ozone during the year. Approximately 67.7 Tg of agricultural residue is burnt over 9593 km^2^ cropland annually^10^, in the N.W. IGP.

Controlled combustion of various biomass fuels under laboratory conditions have revealed presence of greater than 500 different volatile organic compounds (VOCs)^11–13^ differing in their reactivity, health effects and ability to form climate active constituents such as ozone and organic aerosol^14^. Grab sampling experiments which involve collection of ambient smoke plumes directly from the fires in the agricultural fields in pressurized glass flasks/stainless steel canisters followed by subsequent analysis in the laboratory, further allow comparison of laboratory and field determined emission ratios and emission factors for specific kind of biomass fuels^15^. However, the quanta of additional atmospheric reactivity introduced due to such fires^11,12^ as well as the identity and photochemical transformations of the primary emissions in ambient conditions are still poorly understood. In particular, we still do not know whether all important compounds involved in this process have been identified and accounted for in atmospheric models. The oxidizing efficiency of the atmosphere depends primarily on the concentration of ambient hydroxyl radicals (OH)^16–18^. Thus, the presence of additional/unmeasured compounds that can deplete hydroxyl radicals, also has implications for the chemical lifetime of greenhouse gases such as methane, for which the main removal pathway is reaction with hydroxyl radicals^18^. Moreover, gas to particle conversion of reactive gases can influence the growth and properties of cloud condensation nuclei, thereby potentially affecting even the hydrological cycle^19^.

Direct measurement of the total gas phase OH reactivity (mentioned as OH reactivity henceforth) provides a measure of the total gaseous reactive pollutant burden^20^ and fraction of unmeasured reactive VOCs in an atmospheric environment by comparison with the calculated OH reactivity due to the measured OH reactants^21^. The total OH reactivity is defined as the summation over products of the concentration of the OH reactant [X_i_] (e.g. X_i_ = CO, VOCs, NO_2_ etc.) and its rate coefficient with the hydroxyl radical (*k*_*OH*+*Xi*_), expressed mathematically as:

1 $$Total\,OH\,Reactivity=\,{\sum }^{}{k}_{OH+{X}_{i}}[{X}_{i}]$$

This is analytically a very challenging measurement^22^ as there can be thousands of OH reactants in most atmospheric environments^23^. While previous OH reactivity measurements in forested, urban and suburban sites have improved understanding of emissions in these environments^17,21,24,25^, no such investigation of the effects of open agricultural biomass fires using direct OH reactivity measurements in ambient environments exist. Considering the dual effect of fire emissions on production and loss of hydroxyl radicals, their impact on atmospheric oxidation chemistry is still uncertain.

Here, we investigated the impact of agricultural fires by measuring a suite of OH reactants and the total OH reactivity of air directly at a regionally representative suburban site located downwind of agricultural fields in the north-west IGP during the summertime pre-harvest non crop-residue fire influenced and post-harvest crop-residue fire influenced periods from March – May 2013. Detailed analyses of the composition and fraction of missing OH reactivity was undertaken for both periods using a state of the art chemical box model. Further grab sampling and analyses of the chemical emissions from an agricultural biomass fire at the source were also employed for interpretation of the results.

## Results

### Site description and classification of Non Crop-residue Fire Influenced (NCFI) and Biomass Crop-residue Fire Influenced (CFI) periods

**Figure 1:**
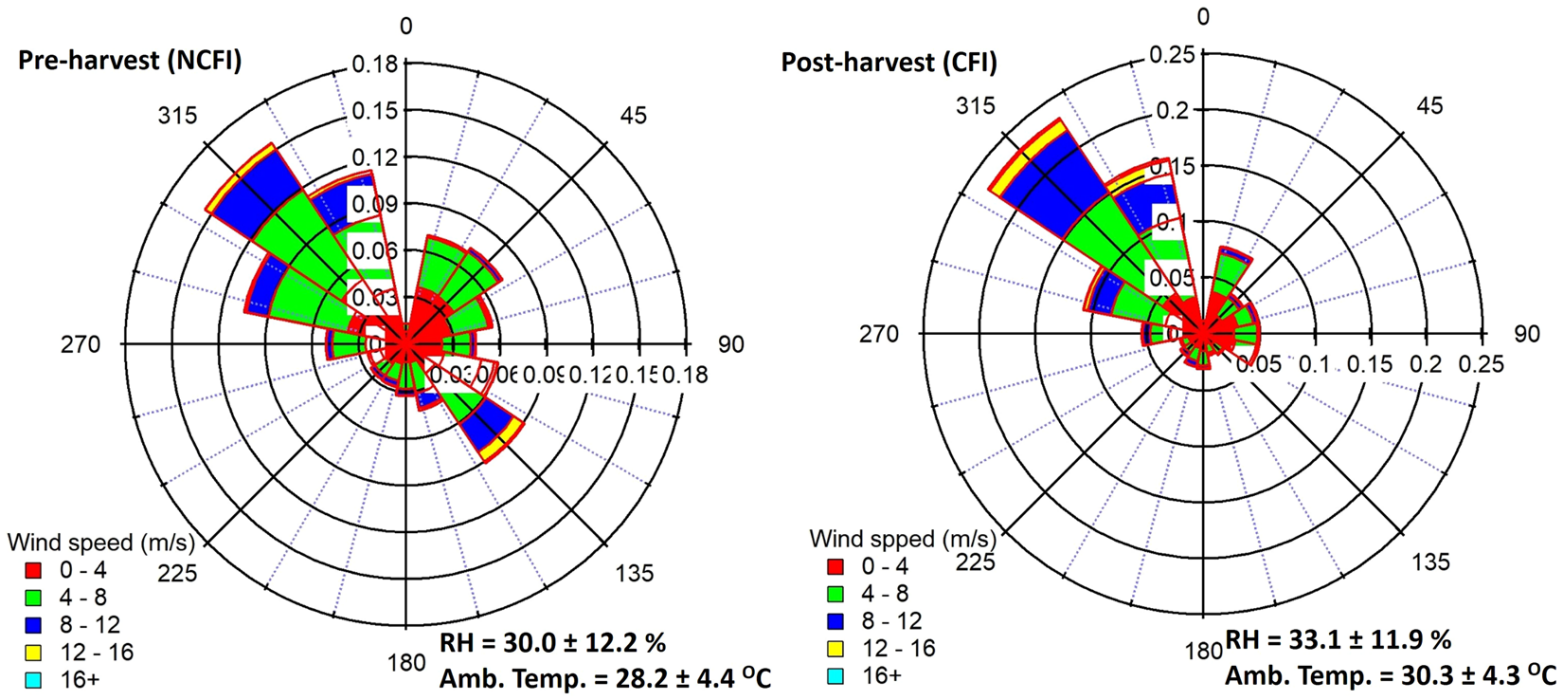
Wind rose plots showing the fractional contribution of different sectors from which airmasses arrived during the pre-harvest NCFI and post-harvest CFI periods.

The ambient VOC and trace gas measurements reported here were performed from 28 February 2013 till 31 May 2013 at the IISER Mohali atmospheric chemistry facility (30.679°N, 76.729°E; 310 m a.s.l.), a regionally representative suburban site located in the north-west Indo-Gangetic Plain (N.W. IGP), which has been described in detail in previous works^26,27^. The three-month long period in 2013 captured the summertime pre-harvest and post-harvest conditions in the N.W. IGP. The year 2013 was not anomalous in terms of emissions and meteorological conditions compared to other years as demonstrated through analyses of multi-year chemical and meteorological data from the site^9^. Ambient total OH reactivity and VOCs were measured using the VOC-OHM (Volatile Organic Compounds – OH reactivity Measurement) system described and characterized in detail in Kumar and Sinha^28^. This technique enables rapid sequential measurements of both ambient VOC concentrations and total OH reactivity at a temporal resolution of 1 minute. The VOC and OH reactivity measurement modes are alternated every ~13 minutes using the same proton transfer reaction mass spectrometer (PTR-MS). The schematic and flow layout for sequential measurements of VOCs and OH reactivity can be found in Fig. 1 of Kumar and Sinha^28^. Details pertaining to VOC measurements using the PTR-MS technique can be found in several comprehensive reviews^29,30^ and details of the OH reactivity technique can be found in previous works^22,31^. While most of the technical details are provided in the methods section, some pertinent aspects are provided below.

VOC measurements using PTR-MS are based on the principle of “soft” chemical ionization mass spectrometry in which hydronium ions (H_3_O^+^) act as the primary reagent ions within the instrument. VOCs having a proton affinity greater than that of pure water (165.2 kcal mol^−1^) can typically be detected at a nominal mass to charge ratio (m/z) equal to their molecular weight+1, generally without fragmentation for small organic ions. A PTR-MS equipped with a quadrupole mass analyzer (PTR-QMS) cannot resolve nominal isobaric ions with its unity mass resolution and hence identification of compounds based only on the m/z values can in principle result in overestimations of the true ambient concentrations of compounds. However, reviews^29,30^ of several studies and inter-comparisons between PTR-MS and other more specific analytical techniques (e.g. gas chromatography based instruments) in diverse environments and plumes have shown that for compounds such as methanol, acetonitrile, acetaldehyde, acetone, methyl ethyl ketone, sum of C-9 aromatics and sum of methyl vinyl ketone and methacrolein, only minor contributions from other isobaric compounds have been observed in ambient atmospheric environments. Hence one can be confident of the compound assignment for these species using PTR-QMS. The potential interferences in the mass identification of rarely measured VOCs using PTR-QMS are discussed later (Methods).

Direct OH reactivity measurements were performed during two measurement intensives: from 10 April 2013 till 21 April 2013 to quantify the pre-harvest summertime OH reactivity and from 11 May 2013 till 17 May 2013 to quantify the post-harvest summertime OH reactivity. The most prevalent wind direction at the site was from the north-west (Fig. 1), where the land use is primarily agricultural and rural in nature^26^ and wheat crop is grown extensively (~3.5 million hectares in the state of Punjab)^32^. The black circle in Supplementary Fig. 1 shows the location of the site. The N.W. IGP is influenced by the biomass crop residue fires in May, a practice that gained popularity with the introduction of mechanized combine harvesters in 1986. Satellite fire counts data over the region are shown as detected by the Moderate Resolution Imaging Spectroradiometer (MODIS)^33^ installed on two sun-synchronous polar orbiting satellites called Aqua and Terra (red dots in Supplementary Fig. 1). The start and end periods of post-harvest wheat residue fires could be ascertained from the daily satellite fire counts data (shown in inset of Supplementary Fig. 2) and were found to be consistent with enhancements in chemical tracers of biomass fires such as acetonitrile (see Fig. 4; Supplementary Table 1 and Supplementary Fig. 3)^9^. In the spatial extent of fires observed by the MODIS satellite, large scale fires are only observed in the states of Punjab and Haryana which majorly consist of irrigated croplands. Significant fires are not observed in the states of Jammu & Kashmir, Himachal Pradesh and Uttarakhand which comprises of forested regions^27^, so the occurrence of large scale forest fires during our study period is highly unlikely. As no major highway or industry was reported to have been commissioned in the fetch region upwind of our measurement site^9^ during the study period, it is also highly unlikely that the traffic related emission sources changed considerably between pre-harvest and post-harvest summertime periods. On the other hand, the crop residue fires were visible regionally, widespread and could be discerned both using satellite fire activity data as well as chemical tracer compounds characteristic of biomass burning.

Figure 1 shows the wind roses indicating the fetch region for the pre-harvest and post-harvest periods reported in this work along with the temperature and relative humidity values (average ± 1σ ambient variability). The most frequent fetch regions, temperature and relative humidity were similar for both periods. Clear sky conditions were observed during both pre and post-harvest measurement periods except the period from 11 May 2013 (afternoon) till 12 May 2013 (noontime) when cloudy conditions and 16 mm of rainfall occurred. This can also be observed in the solar radiation data shown in Supplementary Fig. 2. To ensure that such washout of pollutants due to rain do not drive the differences between the two measurement periods, the periods with overcast condition and rainfall (shown as blue shaded region in Supplementary Fig. 2) were excluded from further analyses. Consistency of fetch region and meteorological conditions implied that the key difference between both periods were the chemical composition changes due to agricultural biomass fire emissions.

Henceforth, we refer to the period from 10 April 2013 to 21 April 2013 as the pre-harvest Non Crop-residue Fire Influenced period (NCFI) and the period from 11 May 2013 to 17 May 2013 (except 11 May 2013 15:30 to 13 May 2013 11:30 local time) as the post-harvest biomass Crop-residue Fire Influenced period (CFI). These two periods were representative of the extended pre-harvest summertime conditions when the biomass crop residue fires were absent (28 February 2013 to 30 April 2013; termed extended NCFI period) and the post-harvest biomass crop-residue fire influenced period (01 May 2013 to 31 May 2013; termed extended CFI period), respectively. This is also borne by the similarity in the diel concentration profiles derived from measurements performed during the intensives (Supplementary Fig. 3) and profiles of the same compounds, namely acetonitrile, sum of C-9 aromatics, acetaldehyde and acetone, for extended NCFI and extended CFI periods as shown later.

### Diel variability and chemical composition analyses of total and calculated OH reactivity

**Figure 2:**
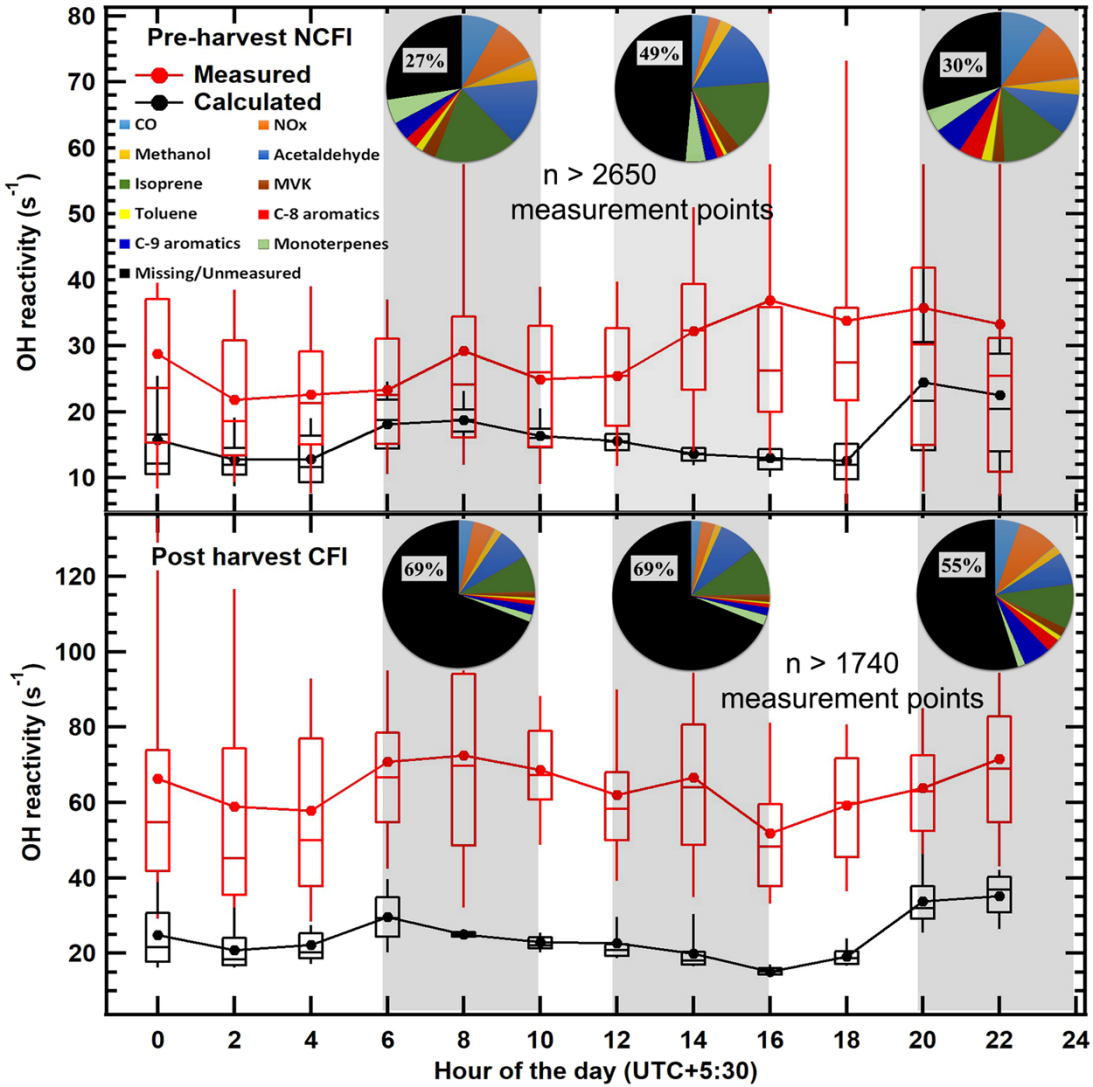
Diel box (25^th^–75^th^ percentiles) and whiskers (10^th^, 90^th^ percentiles) concentration profiles of the directly measured total OH reactivity and the calculated OH reactivity due to measured species in the NCFI period (top) and CFI period (bottom). The average and various percentiles of the data in between a given 2 hour time interval are plotted against the start hour of the interval. The pie charts show partitioning of the measured OH reactivity among top ten measured OH reactants in 4-hour windows (shaded in the figure) of the morning (06:00–10:00 Local Time (L.T.)), afternoon (12:00–16:00 L.T.) and evening time (20:00–24:00 L.T.).

Figure 2 shows the magnitude and diel variability of the total measured OH reactivity (red) and calculated OH reactivity (black) due to measured OH reactants for both NCFI and CFI periods. The calculated OH reactivity shown in Fig. 2 was calculated according to equation (1) using measured concentration of OH reactants listed in Supplementary Table 1 including O_3_ and SO_2_. The relevant rate constants for reaction of individual OH reactants with hydroxyl radicals were taken from Atkinson *et al*.^34,35^. The pie charts in Fig. 2 further show the contribution of individual OH reactants and the fraction that could be accounted for by the measured compounds. Both the measured and calculated OH reactivity (OHR) increased significantly during CFI periods relative to NCFI periods, with greater than 120 % increase in the average measured OH reactivity (28 s^−1^ to 64 s^−1^), accompanied by 40 % increase in the average calculated OH reactivity (15 s^−1^ to 21 s^−1^). The missing OH reactivity was the highest in the afternoon (49 %) followed by evening (30 %) and morning (27 %) hours during the NCFI period. However, during the CFI periods, surprisingly the unaccounted fraction of OH reactivity was always higher than that accounted for by the measured OH reactants ranging from as high as 69 % during morning and noon hours to 55 % in the evening hours. This suggests the presence of many new/unmeasured VOCs during the CFI periods, with likely significant contribution from oxidation products. Typically agricultural burning of the wheat straw occurs in the evening or early morning hours^36^ resulting in large contribution of primary emissions to pollutant loadings. The contribution of non-oxygenated VOCs (isoprene, aromatics, monoterpenes) and carbon monoxide (CO) in the CFI period was less than 27 %, and the maximum occurred during evening hours. Higher variability was also observed in the directly measured total OH reactivity during the CFI period (Supplementary Fig. 2), consistent with rapidly changing VOC concentrations in sampled pollution plumes^37^. Supplementary Table 1 lists the average, range of peak concentrations (75^th^–90^th^ percentiles) and enhancements which occurred at a confidence interval greater than 99.9 % for the key measured OH reactants including acetonitrile, a chemical tracer of biomass-fires. Enhancement ratios ranging from 1.5–2.0 were observed for several reactive VOCs such as methyl ethyl ketone, isoprene, acetaldehyde, and the sum of C-9 aromatic compounds (e.g. trimethylbenzene). Supplementary Table 2 similarly lists the enhancement ratios of additional reactive compounds measured in the same season during the extended NCFI and CFI periods, which also ranged from 1.5–2.2 for most compounds.

The calculated OH reactivity only accounted for the directly measured OH reactants (total of seventeen gases), which does not include several reactive oxidation products such as pinonaldehyde, formaldehyde, propionaldehyde, glyoxal, methylglyoxal and acrolein which are formed from the measured organic compounds. As oxygenated VOCs are known to contribute significantly towards OH reactivity of biomass burning plumes, it is important to account for them in the OH reactivity calculation^11^. Further, several compounds known to be emitted from biomass fires such as C2-C5 alkanes/alkenes and their oxidation products were missing from the set of directly measured OH reactants. To account for the contribution of all these important species that were not directly quantified, we therefore applied a detailed 0-D steady-state box model^38^ employing MCM 3.3.1 chemistry containing 1363 species^39^, for analyzing the OH reactivity budget in both NCFI and CFI periods. The model was constrained with diel hourly average concentrations of seventeen measured chemical species, methane and hydrogen during respective periods, twelve approximated C2-C5 alkanes and alkenes (Supplementary Tables 1–3) and meteorological parameters (Methods). For estimating the concentrations of C2-C5 alkanes and alkenes which were not directly measured during our study (Supplementary Table 3), we multiplied the hydrocarbon/CO emission ratios reported by Andreae and Merlet^3^ in agricultural residue fires with the average CO concentration measured during the CFI period. The hydrocarbon concentrations during the NCFI periods were then estimated by dividing the CFI period concentrations by 1.6, which was the average enhancement ratio of the directly measured VOCs between the CFI and NCFI periods.

**Figure 3:**
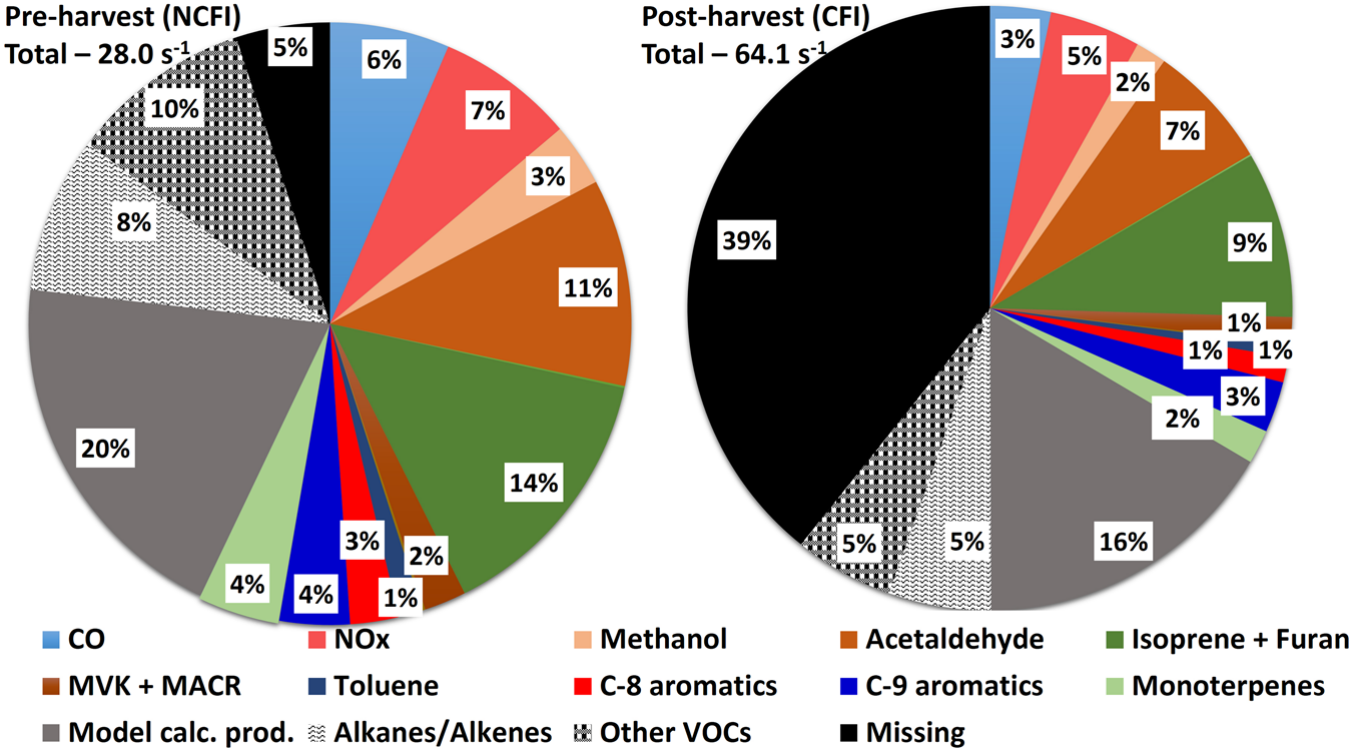
Pie charts showing the missing OH reactivities, and average contribution of measured OH reactants, estimated hydrocarbons and model calculated oxidation products to the total measured OH reactivity for (**a**) NCFI and (**b**) CFI periods.

Figure 3 summarizes the results which show that the average total OH reactivity in the NCFI period can be fully explained (~95%) by the compounds present in the model (within measurement uncertainty of ~20 %), whereas using the same set of OH reactant compounds and chemistry, the model is able to explain only ~60 % of the total OH reactivity during the CFI periods. Thus a large unexplained missing OH reactivity fraction of ~40% persists. We note that percentage contribution of the modeled oxidation products changed from 20% of 28 s^−1^ which is 5.6 s^−1^ in NCFI period to 16% of 64 s^−1^, which is 10.2 s^−1^ in CFI period (Fig. 3). So in terms of absolute magnitude the contribution increased significantly (by 82%) relative to the NCFI period. This points to the increased contribution of photochemically formed reactive compounds to the total measured OH reactivity during the CFI periods. Increased initial concentrations of measured compounds definitely led to higher concentration of the model calculated oxidation products.

For many of the rarely measured VOCs (Supplementary Table 2), a calibration gas standard was unavailable and so the total uncertainty of the measurement for these VOCs is ~30%. To assess how much the 30 % concentration uncertainty for rarely measured VOCs could affect our OH reactivity calculations and hence our conclusion of the large missing OH reactivity during the CFI periods, model runs were performed for the NCFI and CFI periods with 30 % higher concentrations of rarely measured VOCs compared to the actual measurements. We found that doing so reduced the missing OH reactivity fraction only to 3 % (from 5 %) in the NCFI period and to 37 % (from 39 %) in CFI period. Model sensitivity runs were also performed assuming minimum and maximum rate constants for the lumped species (e.g. sum of C-8 aromatics, sum of C-9 aromatics and sum of monoterpenes) to provide upper and lower bounds (assuming the least reactive or most reactive compound contributed to the entire measured m/z) of the missing OH reactivity (Supplementary Note 1). The change in values of missing OH reactivity relative to present values reported in Fig. 3 (5 % missing in NCFI and 39 % missing in CFI), still resulted in no missing OH reactivity in NCFI period (within uncertainty of measurements) and less than 6 % reduction in missing OH reactivity for the CFI period. Thus, the conclusion concerning a large fraction of unexplained OH reactivity in the CFI period remained unaffected. For estimating the concentrations of C2-C5 alkanes and alkenes which were not directly measured, we did not subtract the background CO concentrations. Generally enhancements in CO are used to estimate concentrations of hydrocarbons when multiplying by the VOC/CO emission ratios. In order to assess the magnitude of change in missing OH reactivity due to this, we undertook the following calculation. Given that the measured average CO in CFI period was 395 ppb (Supplementary Table 1) and the 10^th^ percentile of CO data (reasonable to consider as the background CO) in CFI period was 150 ppb, the resulting increase in missing OH reactivity due to lower approximated C2-C5 alkanes/alkenes concentrations, was found to be less than 3% in CFI period and less than 2% in NCFI period. Thus, no significant bias arises in the missing OH reactivity fraction during CFI and NCFI periods due to the current method of estimating the C2-C5 hydrocarbon concentrations. In addition to the C2-C5 alkanes and alkenes, larger alkanes/alkenes and furans are known to be emitted from agricultural residue fires^3^. Using the emission ratio with respect to CO and the concentration enhancement in CO, we have estimated the concentration and subsequently the OH reactivity of 4-methyl-1-pentene, 1-hexene, n-hexane, isohexane, heptane, octene, 2-methylfuran, ethylfuran, dimethylfuran, tetrahydrofuran, benzofuran and furfural in the CFI period. The total OH reactivity due these species during the CFI period is 0.35 s^−1^ which is ~0.5% of the directly measured total OH reactivity. Again the missing OH reactivity fraction remains virtually unchanged.

**Figure 4:**
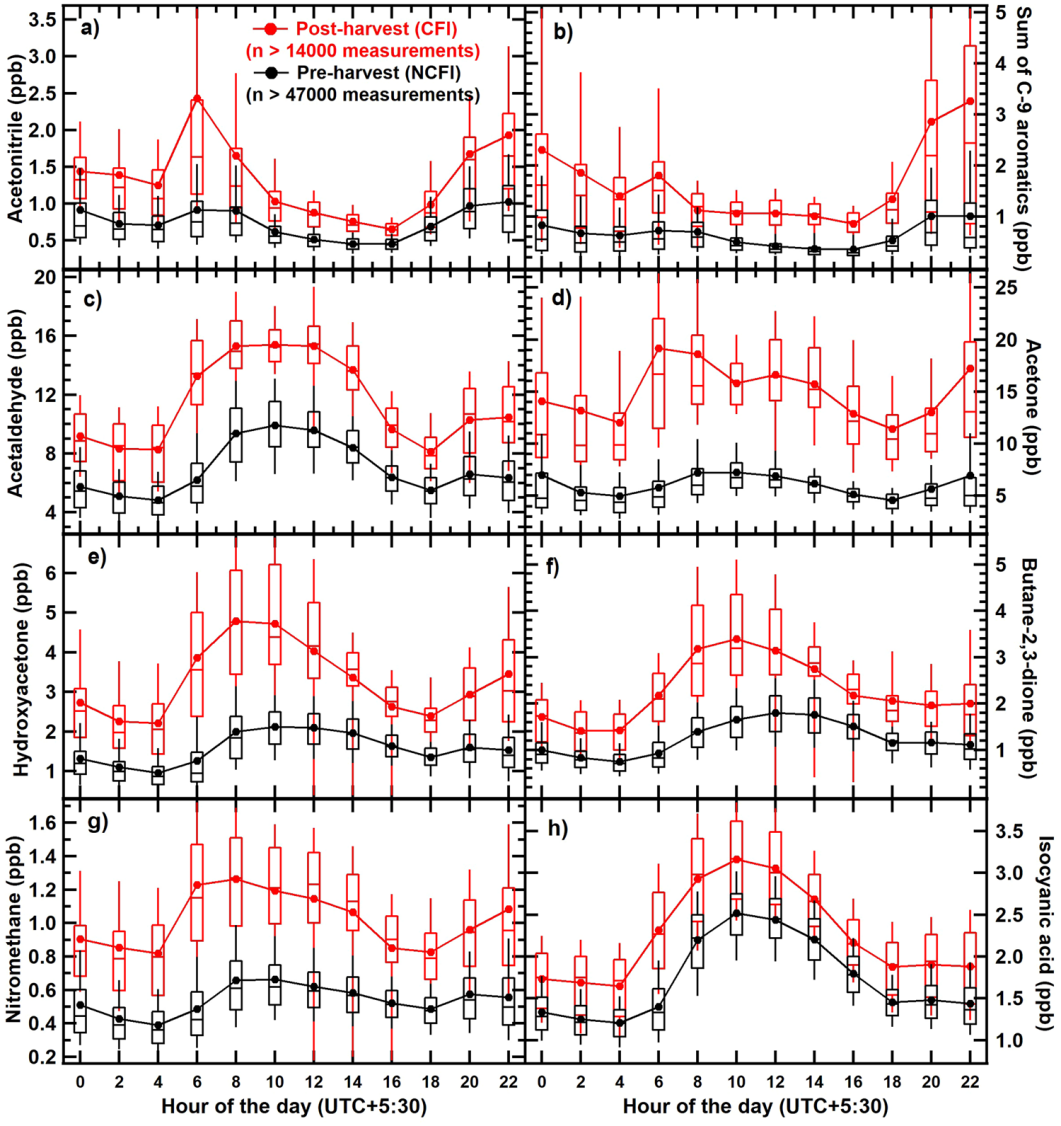
Diel box (25^th^–75^th^ percentiles) and whiskers (10^th^, 90^th^ percentiles) profiles of the measured concentrations of (**a**–**d**) acetonitrile, sum of C-9 aromatics, acetaldehyde and acetone, in the CFI (red) and NCFI (black) periods and (**e**–**h**) hydroxyacetone, butane-2,3-dione, nitromethane and isocyanic acid in the extended CFI and NCFI periods of summer 2013.

Figure 4 shows the strong enhancements that occurred during the CFI period in the concentrations of acetonitrile (a chemical marker compound for biomass-burning), sum of C-9 aromatics (trimethylbenzenes), as well as acetaldehyde and acetone which are emitted from biomass fires but are also formed photochemically due to oxidation of precursor compounds emitted from the fires such as C2-C5 alkanes and alkenes^40^. Surprisingly, we also observed much higher photochemical formation rates in rarely measured VOCs like hydroxyacetone and butane-2,3-dione and strong enhancements in ambient concentrations of photochemically formed nitrogenous compounds e.g. nitromethane and isocyanic acid (HNCO) during the CFI periods. The slope of the line fit from sunrise (05:00 local time) till attainment of peak daytime concentrations for hydroxyacetone increased from 0.27 ppb h^−1^ in the NCFI period to 0.54 ppb h^−1^ in the CFI period whereas that of butane-2,3-dione increased from 0.19 ppb h^−1^ to 0.43 ppb h^−1^ between NCFI and CFI periods. The removal of HNCO from the atmosphere takes place primarily through wet and dry deposition, since the two major gas phase pathways, reaction with hydroxyl radical (OH) and photolysis are slow^41^. The solubility of HNCO and its kinetics imply lifetimes due to heterogeneous removal on the order of days to a few weeks^42^, which would be even slower under the extremely dry conditions of the Indian pre-monsoon summer season (RH as low as 15% in the daytime^9^). Thus HNCO would have a high accumulation tendency under such conditions. The precursor of isocyanic acid through hydroxyl radical initiated oxidation is formamide^41^ which in turn can be formed from hydroxyl radical initiated oxidation of methylamine (Supplementary Note 2). Further, formation of nitromethane can occur from ozone initiated oxidation of dimethylamine. With the PTR-MS technique dimethylamine, ethylamine and formamide can be detected at m/z 46 after protonation. Similarly trimethylamine and acetamide can be detected at m/z 60 after protonation. Since dimethylamine, ethylamine and trimethylamine cannot be formed photochemically, we attributed the signals to be majorly due to oxidation products of amine oxidation i.e. formamide at m/z 46 and acetamide at m/z 60 for the daytime data (Supplementary Fig. 4). However for the night time data, there could certainly be equally significant contribution from both the alkylamines and amides. The diel variation of m/z 46 and m/z 60 (Supplementary Fig. 4) measured with the PTR-MS clearly show strong evening time secondary peaks only during the CFI periods. These peaks would coincide very nicely with the timing of the fire activity (evening). Further it is known from laboratory studies and has been reported by Stockwell, *et al*.^12^ (Supplement) that burning of many agricultural residues (e.g. African grass, wheat residue, paddy residue, alfalfa, hay, sugarcane and sawgrass) emits several alkylamines. It appears that higher concentrations of both amines and amides emitted at night during CFI periods tend to accumulate and just after sunrise trigger photochemical production of the oxidation products such as amides, nitromethane and isocyanic acid. As the concentrations of the precursor compounds are higher in the CFI periods, the rate of photochemical formation which depends on the concentration of the precursor compounds is also higher in CFI period.

## Discussion

### Potential contributors to the missing OH reactivity

**Figure 5:**
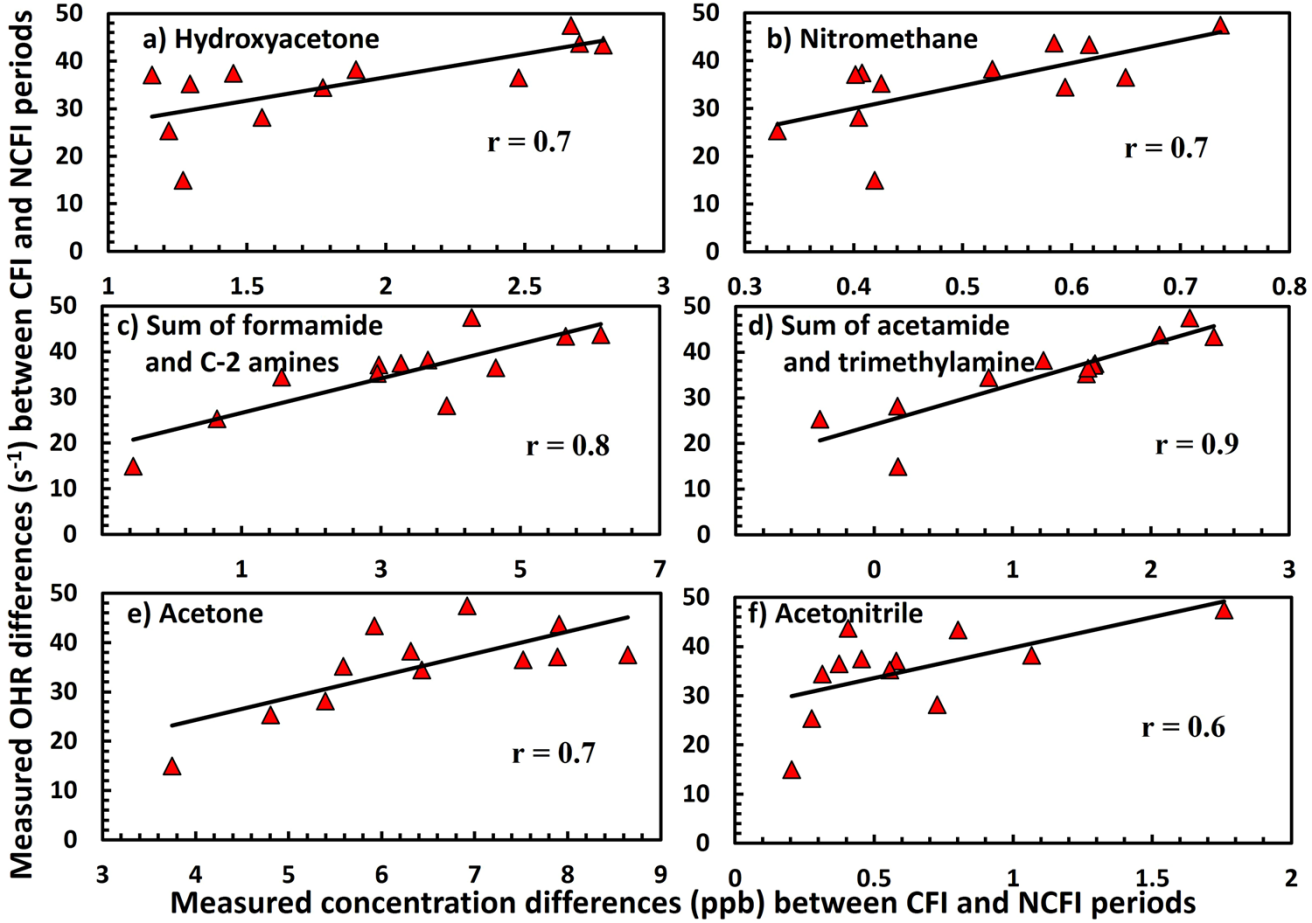
Linear regression lines showing correlation of the increased OH reactivity between CFI and NCFI periods with the absolute concentration increases between CFI and NCFI periods of (**a**) hydroxyacetone, (**b**) nitromethane, (**c**) Sum of formamide and C-2 amines, (**d**) Sum of acetamide and trimethylamine, (**e**) acetone and (**f**) acetonitrile (biomass burning tracer). The individual points in the different panels were obtained by calculating the differences between CFI and NCFI periods using the average diel profile data for measured OH reactivity shown in Fig. 2 and hydroxyacetone, nitromethane, acetone and acetonitrile shown in Fig. 4 and Sum of formamide and C-2 amines (m/z 46), Sum of acetamide and trimethylamine (m/z 60) shown in Supplementary Fig. 4.

Hydroxyacetone and butane-2,3-dione are formed primarily as photo-oxidation products of isoprene and 1,2,4-trimethylbenzene^39,43^ with some direct emission also having been observed from agricultural crop residue fires in laboratory studies^12^. The photochemical formation of nitromethane is likely driven by ozone initiated atmospheric oxidation of compounds such as trimethylamine which also has high OH reactivity (Table 1). Significant photochemical formation of formamide and acetamide was also observed in the daytime data during CFI period. The photochemical formation of several first and second generation photochemical oxidation products of amines e.g. formamide, acetamide, nitromethane and isocyanic acid strongly suggest that their precursor compounds such as alkylamines are also being emitted from crop residue fires. Both agricultural biomass fires and animal husbandry activities^12,44^ are recognized as major atmospheric sources of alkylamines. Considering that the fetch region was similar during both the NCFI and CFI periods, and there was no evidence for substantial increase in animal husbandry activities between the two periods, we surmise that the agricultural biomass fires are a likely source for the emission of alkylamines. While the OH reactivity contribution of nitromethane, isocyanic acid, butane-2,3-dione and hydroxyacetone is collectively less than 2 s^−1^ in the CFI period, their reactive precursors such as alkylamines have very high OH reactivity (see Table 1) and could help explain a significant fraction of the missing OH reactivity. Figure 5 shows the correlation of the increased OH reactivity between CFI and NCFI periods with absolute concentration increases between CFI and NCFI periods of photochemically formed and nitrogen containing compounds such as hydroxyacetone, nitromethane, sum of formamide and C-2 amines, sum of acetamide and trimethylamine, acetone and acetonitrile. The individual points in the different panels were derived by calculating the differences between CFI and NCFI periods using the average diel profile data of measured OH reactivities (shown in Fig. 2), hydroxyacetone, nitromethane, acetone and acetonitrile (shown in Fig. 4) and the data for sum of formamide and C-2 amines (m/z 46), sum of acetamide and trimethylamine (m/z 60) shown in Supplementary Fig. 4. Acetonitrile being a chemical marker for biomass burning is a proxy for primary fire emissions and its good correlation with the enhanced OH reactivity observed during CFI periods indicates that primary fire emissions which could not be measured likely contributed to the high OH reactivity in CFI period. The highest correlation (r ≥ 0.8) was observed for sum of formamide and C-2 amines (m/z 46), sum of acetamide and trimethylamine (m/z 60), suggesting that nitrogen containing compounds may be playing a key role in driving the enhanced OH reactivity during the CFI period. Further, the good correlation (r ≥ 0.6) of the enhanced OH reactivity in the CFI periods with hydroxyacetone, nitromethane, and acetone, all compounds which were observed to have a strong photochemical source in our measured dataset, also suggests that oxidation products of precursor compounds emitted from the fires contributed to the high measured OH reactivity in the CFI period. Based on chamber oxidation studies of OH radical initiated oxidation of trimethylamine, 1 ppb of trimethylamine can yield 0.75 ppb of formaldehyde (HCHO) and trace amounts of less reactive nitrogen containing VOCs (amides and N-nitroamines)^45^. Using the product yields and the relevant rate constants for reaction of these oxidation products with OH radicals, the presence of an additional 14 ppb of trimethylamine like reactive compounds and the extra photo-oxidation products^45^ formed thereof, could account for the entire missing OH reactivity fraction of ~40% (25 s^−1^) observed during the CFI period. While we are not suggesting that a high concentration of 14 ppb of only trimethylamine would be present in ambient air, the point is that given that dimethylamine, ethylamine and methylamine have rate constants with the hydroxyl radical which are of the same order of magnitude (10^−11^ cm^3^ molecule^−1^s^−1^; Table 1), and the observed diel profiles of m/z 46 and m/z 60 show values as high as 12 ppb and 3 ppb at night (m/z at which methylamine and ethylamine can contribute significantly at night), and the consideration that approximately 150 amines have been detected in ambient air^44^ with smoke being a major source of amines, it is plausible that collectively amines could explain a major fraction of the missing OH reactivity in the crop residue fire impacted periods.

**Table 1:** Rate constants (cm^3^ molecule^−1^ s^−1^) of alkylamines for reaction with OH radicals and O_3_^67,68^.

Alkylamine	k_OH(298)_	k_O3(298)_
Methylamine	2.20E-11	2.13E-20
Ethylamine	2.77E-11	2.76E-20
Dimethylamine	6.54E-11	2.61E-18
Trimethylamine	6.09E-11	9.73E-18

Grab sampling of wheat residue fires in a nearby agricultural field (30.652° N, 76.709° E, 305 m a.s.l.) under ambient smoldering (low modified combustion efficiency (MCE)) and flaming wheat straw fire stages yielded extremely high emission factors of several compounds during the smoldering stage of the fire (Table 2). Biomass combustion experiments conducted in laboratory studies^11^ are based primarily on data from flaming stage fires. In the real world, such smoldering fires can persist for hours and the “poor” combustion conditions are rarely captured and characterized in laboratory studies. Considering that the temperatures are really high (~40 °C)^9^ during May in north-west IGP, in order to keep fires from spreading out of control, we noted that farmers do sprinkle water at the border of certain patches in their field. This practice could certainly contribute to smoldering fires characterized by low combustion efficiency at-least for several patches in a large agricultural field. Moreover the straws are less than 1 foot above the ground and root-bound at the time of fire, which can contribute to a lowering of the MCE. As the VOC emission factors of flaming stage fires are significantly lower than the smoldering stage, we believe our grab sampling experiment results support the contention that several compounds may be underestimated/unreported as emissions from biomass fires. Additionally, the high reactivity due to photochemically formed pollutants in the ambient atmospheric environments would escape notice in the laboratory studies focused on characterizing the primary emissions. The relevance of the reported emission factors for the smoldering stage fires in our study is that they demonstrate that several compounds with negligible emission factors in the flaming stage can have really high emission factors in the smoldering stage. As we could not sample statistically significant number of fires, it would be inappropriate to use them as representative values, but they are sufficient evidence for the primary point, that the reduced nitrogen compounds can be emitted in large quantities and this is the new insight of the emission factor data reported here.

**Table 2:** Emission factors (grams of VOC emitted per kg of dry fuel weight burnt) of different VOCs obtained from source sampling of emissions from open burning of wheat residue during flaming and smoldering stages^15^ in the agricultural field at Raipur Khurad, Mohali (30.652° N, 76.709° E, 305 m a.s.l.), India.

m/z	Major contributors	Emission factor (g/kg of fuel burnt)	
		Flaming MCE = 0.99	Smoldering MCE = 0.43
33	Methanol^74^	0.2	41.3
45	Acetaldehyde^12^	0.2	23.3
97	2-Furaldehyde, 2,5-Dimethylfuran^12^	0.1	16
75	Hydroxyacetone^12^	0.2	15.9
59	Acetone^12^	0.07	13.2
69	Isoprene/furan^49^	0.09	11.5
87	Butane-2,3-dione^12^	0.2	10.7
61	Acetic acid^49^	1.9	9.2
57	Acrolein/1-Butene^12^	0.1	7.8
73	MEK^12^	0.05	6.2
71	MVK + MACR^12^	0.04	5.2
42	Acetonitrile^12^	0.02	4.4
93	Toluene^49^	0.03	2.8
79	Benzene^49^	0.05	1.6
107	Sum of C8-aromatics^12^	0.02	1.5
46	Formamide/Dimethylamine/Ethylamine^49^	0.02	0.6
60	Acetamide/Trimethylamine^49^	<0.01	0.6
121	Sum of C9-aromatics^49^	0.01	0.6
105	Styrene^12^	0.01	0.4
31	Formaldehyde^49^	0.01	0.2
44	Isocyanic acid^49^	0.03	0.2
63	Dimethylsulphide^52^	0.01	0.2
137	Monoterpenes^12^	0.002	0.2
47	Ethanol/formic acid^49^	0.01	0.1

The number in the superscript provides reference to a study in which these compounds were detected in agricultural residue fire plumes.

### Implications for regional air quality and climate

**Figure 6:**
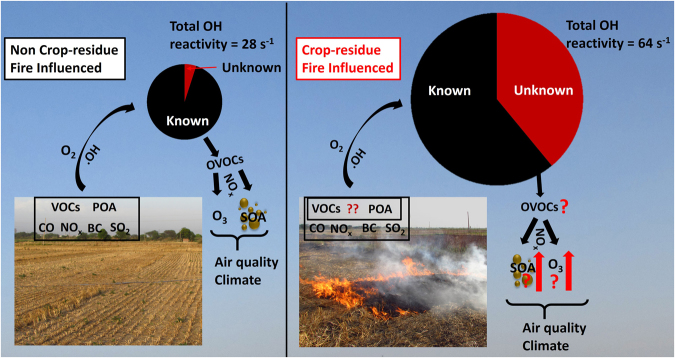
Diagram summarizing effects on air quality and climate relevant processes due to the large unknown suite of reactive compounds emitted directly and formed photochemically as a result of large scale agricultural crop-residue fires. Total OH reactivity provides a measure of total gaseous reactive pollutants directly emitted and formed via atmospheric oxidation of primary emissions even in absence of speciated emission measurements. The radius of the pie chart is scaled as per the observed total measured OH reactivities. POA, BC and SOA stand for primary organic aerosol, black carbon and secondary organic aerosol respectively. The photographs of the agricultural fields were taken by B.P. Chandra at Raipur Khurad, Mohali.

Total OH reactivity provides a measure of all gaseous reactive pollutants that are directly emitted and formed by atmospheric oxidation of the emissions. The large missing OH reactivity during periods affected by agricultural fires has major implications for understanding the formation of tropospheric ozone and other secondary pollutants in biomass fire impacted atmospheric environments (Fig. 6). It reveals that in the biomass fire impacted periods almost half of the reactive atmospheric chemical composition is unknown and therefore their chemical feedbacks are not considered in typical models and field studies. For example, atmospheric oxidation of hydroxyacetone forms HO_2_ radicals (Supplementary Note 3), which may be an important atmospheric pathway for reforming hydroxyl radicals and sustaining/enhancing oxidizing efficiency in a polluted environment. It has previously been shown^9^ that the maximum number of exceedance events in the 8h NAAQS for ozone in India (100 µg m^−3^) occurred in the north-west Indo-Gangetic Plain during the month of May, which is strongly affected by the crop residue biomass fires. 29 and 30 out of 31 days in the years 2012 and 2013, respectively were out of compliance^9^. It was also observed in that study^9^, that the ozone enhancement increased from 19 ppb to 28 ppb between pre-harvest and post-harvest summertime air of the north-west IGP for the air masses with high residence time over the burnt agricultural fields.

Several previous studies from the Indo-Gangetic plain region (for example Rajput, *et al*.^46^ and Ram, *et al*.^47^) have also reported direct emissions and secondary formation of carbonaceous aerosol particles such as elemental carbon and organic carbon including polycyclic aromatic hydrocarbons from the agricultural residue fires. Based on the estimates by Rajput, *et al*.^46^ circa 26% and ~7% of the PM_2.5_ mass fraction is organic carbon and elemental carbon respectively in the wheat residue emissions from the IGP region. PM data from IISER Mohali atmospheric chemistry facility^27^, during the period of our study show that between the NCFI and CFI periods, the average mass concentration of PM_2.5_ increased from 57 µg m^−3^ to 100 µg m^−3^ whereas for PM_10_ it increased from 128 µg m^−3^ to 213 µg m^−3^ (Supplementary Fig. 6). Wheat residue fires in the summertime have already been reported to increase the number of NAAQS exceedance events for both PM_10_ and PM_2.5_ in the north-west IGP. Garg, *et al*.^48^ have previously also reported co-emission of black carbon in three wheat residue fire plumes in May 2013. Calculation of the Secondary Organic Aerosol (SOA) production potential due to measured concentrations of toluene, benzene, xylene, isoprene and monoterpenes using the method used in Sarkar, *et al*.^49^, showed a 40% enhancement in SOA production potential due to these compounds between the NCFI (4.1 µg m^−3^) and CFI period (5.8 µg m^−3^). Detailed studies of SOA formation processes in similar biomass fire impacted atmospheric environments, also considering the role of amides and alkylamines which can participate in particle formation and growth processes^50^ may be very important and will require further investigations.

Exposure to elevated concentrations of isocyanic acid has been documented to pose health risks^41^ and it is worth noting that the average hourly concentrations were always >1 ppb during the CFI periods. While alkylamines and amides can cause irritation in eye, skin and respiratory system upon high exposure, atmospheric oxidation of alkylamines in the presence of nitrogen oxides can also produce carcinogens like dimethylnitrosamine and dimethylnitramine (Supplementary Note 2)^44^, which together with benzene, another known carcinogen emitted from the fires could pose enhanced health risks^36,51^.

We therefore conclude that a larger suite of compounds and chemical reactions needs to be considered for improved understanding of atmospheric chemistry-air quality-climate feedbacks in biomass-fire impacted atmospheric environments and incorporating direct OH reactivity measurements would also aid for understanding such important processes globally. Currently even the most detailed state-of-the-art atmospheric chemistry models and the chemical mechanisms used therein (Supplementary Table 4) exclude formamide, acetamide, nitromethane and isocyanic acid and their highly reactive precursor alkylamines (e.g. methylamine, ethylamine, dimethylamine, trimethylamine).

All the major compounds detected in the wheat stubble fires are also emitted from a large variety of biomass fuels burnt across various parts of world^12^, including different types of crop residue, natural grasses and wood. However, the variation in the modified combustion efficiency and fuel type does change the emission factors of the VOCs. Emission factors during the flaming stage are not particularly high for wheat when compared to other fuel types. Thus while designing the ambient studies, it is important to take into account the major known emissions as highlighted in previous works (e.g. Stockwell, *et al*.^12^, Akagi, *et al*.^52^) and also discussed in our work. Emission factor recommendations for model predictions^3,53^ typically use emission factors over the MCE range of 0.80–0.99. In order to accurately account for the emissions from the smoldering stages of biomass fires globally, emission factors at lower MCE^54^, which can be significantly higher for some compounds should also be studied in detail in laboratory chamber studies of fires (if possible) and included in emission inventories used for model predictions.

As shown in this study incorporating direct OH reactivity measurements aids in accounting for the total gas phase reactivity and missing compounds. Future studies in the biomass burning affected regions including direct measurements of OH radicals and major OH radical production pathways in addition to the OH reactivity, could be very interesting also from the perspective of whether “shifts” in chemistry^17,55^ occur and if so determining the net impact on the oxidation capacity of such perturbed atmospheric environments.

## Methods

### Sampling details

All the instrument inlets were co-located at ~20 m above ground^26^. The inlets were made from Teflon tubing and protected from dust using inline Teflon particle filters^26^, which were changed every seven days. The facility is positioned on the rooftop and the short inlets resulted in inlet residence times <8 s. The PTR-MS was housed in a separate room on the first floor and had a longer Teflon inlet line (12 m).

### Measurements of total OH reactivity and VOCs using a PTR-MS

The PTR-MS instrument used in this work has been characterized in detail in previous works from our group^26,36^. The instrument was operated at a drift-tube voltage of 600V, pressure of 2.2 mbar and temperature of 333 K corresponding to E/N ratio of ~135 Townsend. The primary ion was greater than 7.5 × 10^6^ cps (counts per second) and the impurity ions e.g. NO^+^ and O_2_^+^ were less than 0.3% and 3.9% respectively of the primary ion. The dwell time at m/z channels corresponding to the individual VOCs was 1s. The measured counts per second (cps) at each m/z channel was normalized to a total primary ion count of 1 × 10^6^ cps as the sum of the primary (H_3_O^+^) and the first water cluster ions ((H_2_O) H_3_O^+^), drift tube pressure of 2 mbar and drift tube temperature of 298 K. Dynamic dilution of VOC gas standards (Apel-Riemer Environmental, Inc., Colorado, USA) containing a mixture of 12 VOCs at ~500 ppb was used to experimentally obtain the sensitivity in normalized cps/ppb, for methanol, acetonitrile, acetaldehyde, acetone, isoprene, methyl vinyl ketone, methyl ethyl ketone, benzene, toluene, p-xylene, 1,2,4 trimethylbenzene and alpha-pinene^26^. For other compounds reported in this work, the sensitivities were determined theoretically^29^:

2 $$Sensitivity\,(ncps\,pp{b}^{-1})={10}^{-3}\times \frac{{k}_{VOC+{H}_{3}{O}^{+}}L}{{\mu }_{o}{N}_{0}}\times \frac{{N}^{2}}{E}\times \frac{{T}_{VOC{H}^{+}}}{{T}_{{H}_{3}{O}^{+}}}$$

where ${k}_{VOC+{H}_{3}{O}^{+}}$ = Rate constant of proton transfer from H_3_O^+^ to VOC

L = Length of drift tube (9.2 cm)

${\mu }_{o}$ = Reduced mobility of H_3_O^+^ ions (2.8 cm^2^ V^−1^ s^−1^)^56^

N = Number density of gases in the drift tube

E = Electric field across the drift tube

$\frac{{T}_{VOC{H}^{+}}}{{T}_{{H}_{3}{O}^{+}}}$ = Ratio of transmission efficiency of protonated VOC ion and H_3_O^+^ ion

The values of ${k}_{VOC+{H}_{3}{O}^{+}}$ for different VOCs were taken from Zhao and Zhang^57^ and the transmission efficiencies at different m/z channels for our instrument were used for calculation of respective sensitivity factors. We found that the differences between the sensitivity factors derived using calibration experiments and theoretically derived sensitivity factors for the compounds were within 30% of each other. This is consistent with the predicted accuracy of PTR-MS measurements as proton transfer reactions typically occur at the collisional-limit and without specific calibration standards, the accuracy is better than 40%^58^. The detection limit of PTR-MS was typically less than 100 ppt^28^. The measurement uncertainty was less than 16% for all VOCs where experimentally determined sensitivities were obtained through calibrations. Assignment of m/z ratios (Supplementary Tables 1 and 2) to specific compounds was done after careful consideration of the known fragmentation issues at certain m/z, potential isobaric interferences (discussed below), ambient profiles, and changes in instrumental background.

### Potential interferences in mass identification using PTR-MS

#### Butane-2,3-dione

Methacrylic acid, crotonic acid, pentanal, pent-2-one, pent-3-one can interfere with butane-2,3-dione at m/z 87, but these compounds have been reported to cause interferences only in the forested environments^59^. Previous studies reporting PTR-MS measurements from suburban locations or from biomass burning plumes have attributed m/z 87 to butane-2,3-dione only^12,49^.

#### Formamide and acetamide

The signal for acetamide was corrected for 3.4% of acetone contribution at m/z 60 due to the ^13^C isotope^60^. This accounted for ~15–20% correction in acetamide concentration in general. Similarly, the signal for formamide was corrected for the 2.5% contribution of acetaldehyde due to ^13^C isotope at m/z 46.

#### Hydroxyacetone

Butanol isomers, diethyl ether and propionic acid also contribute to m/z 75 when measured with a PTR-MS, but previous studies have found hydroxyacetone to be the main contributor^49,59^.

#### Isoprene

With a PTR-QMS both isoprene and furan are detected at m/z 69. However, with the deployment of the VOC-OHM technique in the month of May at our measurement site, we were able to conclude that the VOC detected at m/z is primarily isoprene^28^. However, even if the contribution of furan at m/z 69 increases due to crop-residue fires, the calculated OH reactivity will decrease due to the lower rate constant of furan (4.01 × 10^−11^ cm^3^ molecule^−1^ s^−1^)^61^ for reaction with hydroxyl radicals. Hence the missing OH reactivity fraction will only increase further and bolster our conclusion that crop residue fires lead to a large unexplained OH reactivity further.

OH reactivity measurements by VOC-OHM technique are based on the comparative Reactivity method^22^. An initial concentration (C1) of a reagent molecule (pyrrole, C_4_H_5_N) is allowed to react with zero air and ambient air respectively in different steps in the presence of synthetically generated OH radicals inside the glass reactor. In the step where only pyrrole, nitrogen and zero air enter the reactor, pyrrole completely titrates OH radicals generated inside the reactor, and its concentration is C2 in this stage. The difference in pyrrole concentration between stage C1 and C2 yields the net available OH radical concentration inside the reactor. When the incoming flow of zero air inside the reactor is replaced by an equivalent flow of ambient air, there is a competition between compounds present in the ambient air and pyrrole for reaction with the net available OH radicals. The pyrrole concentration in this stage (termed C3) is higher than that in the previous stage (C2). Solving for competitive kinetics inside the glass reactor yields the total OH reactivity of the ambient air (${R}_{air}$) which is only dependent on the measured pyrrole concentrations in different steps of the experiment and the rate constant for the reaction of pyrrole with OH radicals (${k}_{p}$).

3 $${R}_{air}=\frac{C3-C2}{C1-C3}.{k}_{p}C1$$

The measurement in the C3 stage is preceded and followed by C2 measurement. Hence for estimation of total OH reactivity using E3, interpolation of the C2 measurements performed before and after the C3 are necessary. In the C3 stage, ~170 sccm of ambient air was sucked into the reactor which was diluted by the total flow through the CRM reactor (~275 sccm). Hence the OH reactivity obtained using Equation (3) was multiplied by the dilution factor (~1.6) to quantify the total OH reactivity of ambient air. Since the total OH reactivity is determined for pseudo first order conditions, a correction factor is applied for slight deviations. This correction factor is calculated numerically^62^ by allowing different concentrations of an OH reactant (e.g. propane) to react with OH radicals at the pyrrole/OH concentration ratio used during the experiments^22,28^. During the two measurement periods reported in this study, the pyrrole/OH ratio varied between 1.8 and 2.7. The accuracy of the method was checked using propane standards of known Reactivity during both the measurement periods. Supplementary Fig. 5 shows the results highlighting good reproducibility, linearity (r^2^ = 0.98) and accountability (slope = 1.06) for the introduced reactivity over the entire dynamic range for experiments performed on 19 April 2013 during the NCFI period and on 17 May 2013 during the CFI period. The maximum introduced reactivity of 91 s^−1^ during these tests actually represent 146 s^−1^ under ambient condition taking into account the dilution inside the reactor. The detection limit for OH reactivity measurements was 4.5 s^−1^ and the total uncertainty was 18.8 %^28^.

OH reactivity measurements performed using CRM method on which our VOC-OHM system is based are known to have interferences due to following two factors:

1. Humidity difference between C2 and C3 steps of measurement which results in different amount of net available OH radicals inside the reactor for reaction with pyrrole and zero air (C2 step) and pyrrole mixed with ambient air (C3 step).
2. OH recycling due to the reaction between HO_2_ and NO in an environment where ambient NO concentrations exceed 10 ppb.

Use of an advanced Gas Calibration Unit (GCU-A, Ionimed Analytik) for generation of zero air directly from the ambient air, matches the humidity to that of ambient air and no significant humidity difference between C2 and C3 steps were observed during our measurements using the VOC-OHM method^28^.

During the measurement periods reported in this work, ambient NO concentrations were generally less than 2 ppb barring seven short peaks in which concentrations reached ~10 ppb (20 April 2013 19:37–19:43, 21 April 2013 14:30–14:35, 13 May 2013 11:48–14:19, 14 May 2013 13:00–13:55, 14 May 2013 06:00–07:30, 16 May 2013 07:50–08:00 and 16 May 2013 23:30–23:40 L.T.). During most of these events, the VOC-OHM system was in C2 step when ambient air was not being sampled or in the ambient VOC measurement mode. The OH reactivity data removed due to NO peaks of 10 ppb was therefore less than ~4.5 % of the total measured OH reactivity data.

### Grab sampling of biomass fire plumes

Samples of plumes from flaming (modified combustion efficiency = 0.99) and smoldering (modified combustion efficiency = 0.43) stages of wheat residue fires were collected in pressurized glass flasks wrapped with aluminium foil^15^ directly at an agricultural field in Raipur Khurad, Mohali (30.652° N, 76.709° E, 305 m a.s.l.) on 2 May 2015 in the evening time (17:30–18:00 local time). Ambient air samples were also collected in separate glass flasks from the field before the fires were lit after ensuring there were no active nearby fires, to establish the background concentrations at the field site. All the whole air samples collected in the glass flasks were then transported and analyzed within 5 hours of sample collection at the IISER Mohali Atmospheric Chemistry Facility using the PTR-QMS instrument in ion selective mode with a dwell time of 1s. The emission factors of VOCs were calculated for flaming and smoldering stages as described in Chandra, *et al*.^15^.

### Ancillary measurements

Carbon monoxide (CO), sum of nitrogen dioxide and nitric oxide (NO_x_), sulfur dioxide (SO_2_), ozone (O_3_) and meteorological parameters such as solar radiation, ambient temperature, relative humidity, wind speed and wind direction were measured continuously at a time resolution of one minute. CO, NO_x_ and O_3_ measurements from the site have already been analyzed and reported from September 2011 to September 2013^9^, along with a detailed description of the sampling methodology and calibration protocol^9,26,51^. Briefly, CO, NO_x_, SO_2_ and O_3_ measurements were performed using gas filter correlation non-dispersive infra-red (GFC-NDIR) technique (Model 48i), chemiluminescence technique (Model 42i), pulsed UV fluorescence technique (Model 43i) and UV absorption photometry (Model 49i; Thermo Fischer Scientific, Franklin, USA) respectively. Data for meteorological parameters were acquired from meteorological sensors (Met One Instruments Inc., Oregon, USA) installed at the measurement site and located within 2 m of horizontal distance from the instrument inlets. CO calibrations were performed on 08 April 2013 and 06 May 2013, NO_x_ calibrations were performed on 26 April 2013 and 22 May 2013, O_3_ calibrations were performed on 28 April 2013 and 23 May 2013 and SO_2_ calibrations were performed on 30 April 2013. The uncertainties in measurements of CO, NO_x_, SO_2_ and O_3_ are less than 6% and the detection limits of these instruments were 28 ppb, 114 ppt, 1 ppb and 1 ppb respectively. Carbon dioxide and methane concentrations in the grab samples were measured using a high precision Cavity Ring Down Spectroscopy (CRDS) analyzer (Model G2508, Picarro, Santa Clara, USA) as described in Chandra, *et al*.^15^. These were used for calculation of emission factors^15^.

### OH reactivity calculations using the Framework for 0-D Atmospheric box Modeling (F0AM) Model

A zero-dimensional atmospheric box model F0AM^38^ was set up for calculating the OH reactivity using the measured and estimated compounds and their oxidation products. The model employs Master Chemical Mechanism (MCM) 3.3.1 chemistry^39,63–65^. The NO_2_ photolysis rate constant J(NO_2_) was calculated using the measured solar radiation^66^. Other model prescribed photolysis frequencies (e.g. J(O(^1^D)), J(HCHO), J(H_2_O_2_), J(HONO) etc.) are corrected using a factor derived using the F0AM “hybrid” method derived J(NO_2_)^38^. The MCM setup included a total of 1363 species and 4205 chemical reactions. The rate constants used in the model are taken from the reviewed rate constants published by Atkinson *et al*.^34,35,67–70^. If unavailable in the former they were taken from the NIST chemical kinetics database^71^. The model was constrained with hourly averaged concentrations of all the directly measured VOCs listed in Supplementary Table 1, VOCs measured in the extended NCFI period (Supplementary Table 2) whose chemistry is present in MCM 3.3.1, O_3_, NO_x_, SO_2,_ J(NO_2_), estimated concentrations of hydrocarbons as described in previous section (Supplementary Table 3), relative humidity and ambient temperature^37,72^. Methane (CH_4_) and hydrogen (H_2_) mixing ratios are held constant at 1.8 ppm and 550 ppb respectively. The model calculated concentrations of secondary species at the end of each hour were used as the initial concentration of model run for the second hour. The simulations are performed in steady state conditions with a spin-up period of three days. A dilution in the background air with zero concentration of all the model calculated species was considered for the model setup^38^. The dilution is parameterized according to the following first order loss equation:

4 $$\frac{d[X]}{dt}={K}_{dil}[[X]-{[X]}_{b}]$$

Here [X] is the concentration of the species and [X]_b_ is the concentration in background air which is assumed to be zero. K_dil_ is the first order dilution constant which is chosen^73^ to be 1/86400 s^−1^ corresponding to an atmospheric lifetime of 24 hours, which was assumed to avoid build-up of unrealistically high concentration of model calculated secondary species. Note that the following measured compounds namely isocyanic acid, formamide, acetamide, 2-furaldehyde, nitromethane and acetonitrile are not included in the MCM 3.3.1, hence the box model calculations do not take into account their chemistry. Also alkylamines, which are precursors of nitromethane, amides and isocyanic acid are not included in the MCM 3.3.1.

### Data Availability

All data can be made available upon reasonable request to the corresponding author at vsinha@iisermohali.ac.in.

## Electronic supplementary material

Supplementary Information
