# Supplementary material for: Large unexplained suite of chemically reactive compounds present in ambient air due to biomass fires
Source: Sci Rep. 2018 Jan 12;8:626. doi: 10.1038/s41598-017-19139-3 (PMC5766614; doi:10.1038/s41598-017-19139-3)
Supplement: Supplementary file 1 — Supplementary Information [file 41598_2017_19139_MOESM1_ESM.pdf]

Large unexplained suite of chemically reactive compounds present in ambient air due to biomass fires

V. Kumar[1], B. P. Chandra[1], V. Sinha[1]*

[1] Department of Earth and Environmental Sciences, Indian Institute of Science Education and Research Mohali, Sector 81, S. A. S. Nagar, Manauli PO, Punjab, India 140306.

*Correspondence to: V. Sinha (vsinha@iisermohali.ac.in)

Supplementary Figure 1:

Temporal and spatial extent of fire counts detected (at ≥ 80% confidence interval) during NCFI (10 April 2013 to 21 April 2013) and CFI periods (11 May 2013 to 17 May 2013) using the MODIS satellite over the north-west Indo-Gangetic Plain. Black circle shows the location of the measurement site (30.667 ^{0}N, 76.729 ^{0}E, 310 m a.s.l.). The map was generated using Panmap software, Version 0.9.6[1] (url : https://doi.pangaea.de/10.1594/PANGAEA.104840)

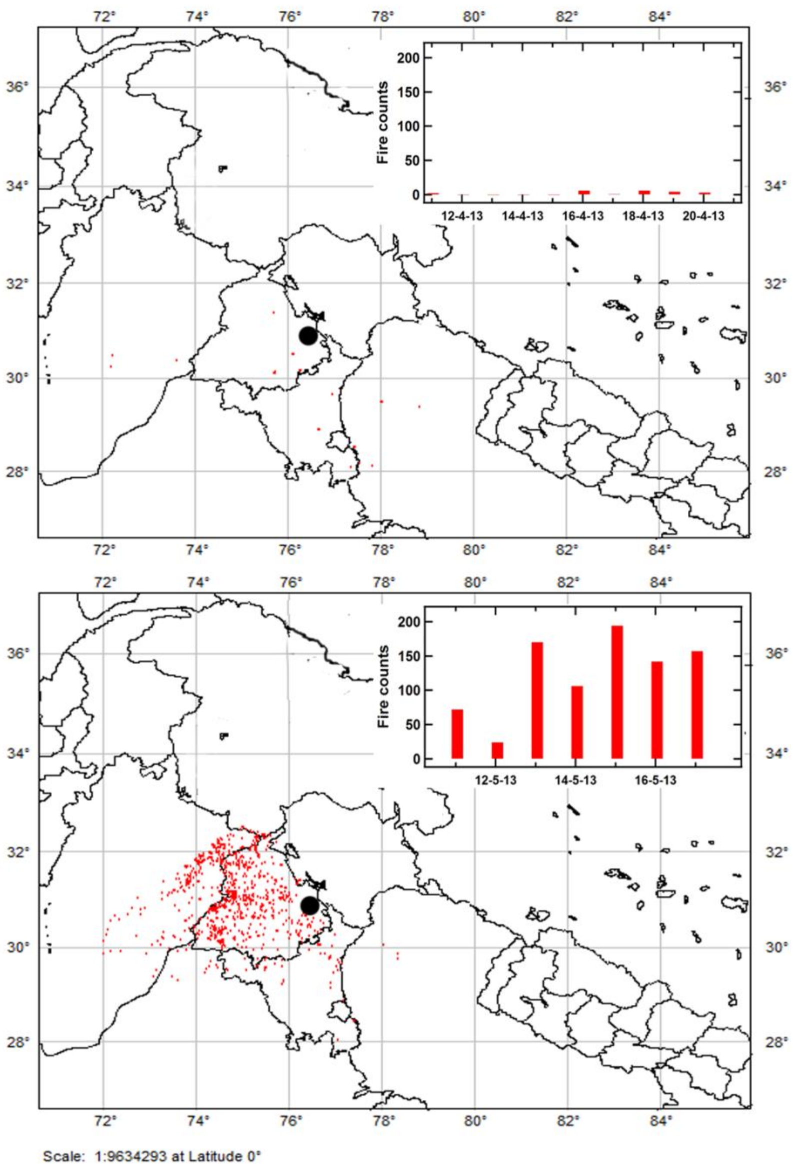

Supplementary Figure 2:

Time series of the measured OH reactivity (3 min temporal resolution) and calculated OH reactivity due to measured compounds (top panel), mixing ratios of acetonitrile, isoprene, acetaldehyde and toluene (second panel) and NO_x and O_3 (third panel). The regional (28°N–33°N lat and 72°E–79°E long) MODIS satellite-derived fire counts and measured solar radiation are shown in the bottom panel. Light green shaded region shows the measurements during pre-harvest NCFI period whereas light pink shaded region shows the measurements

during post-harvest CFI period. The blue shaded region shows periods when overcast conditions and rain were observed.

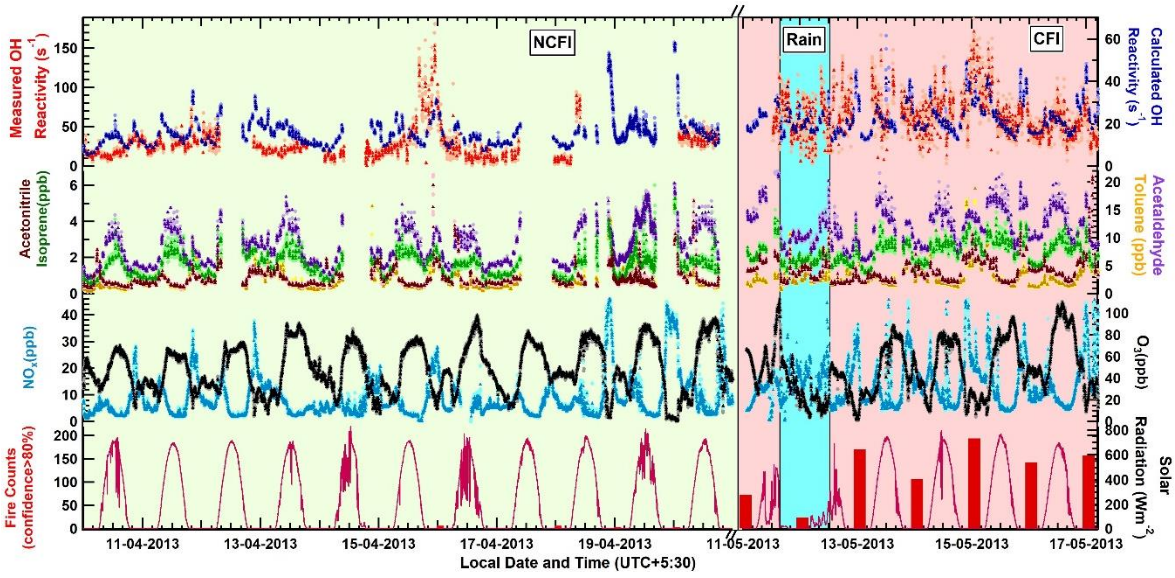

Supplementary Figure 3:

Diel box and whisker plots of measured concentrations (in ppb) of acetonitrile, sum of C-9 aromatics, acetaldehyde and acetone in the pre-harvest (NCFI) and post-harvest (CFI) period of summer 2013. The box represents inter-quartile range (25th-75th percentile) whereas the whiskers represent 10th and 90th percentiles. Average and median are shown as solid circle and dashed line, respectively.

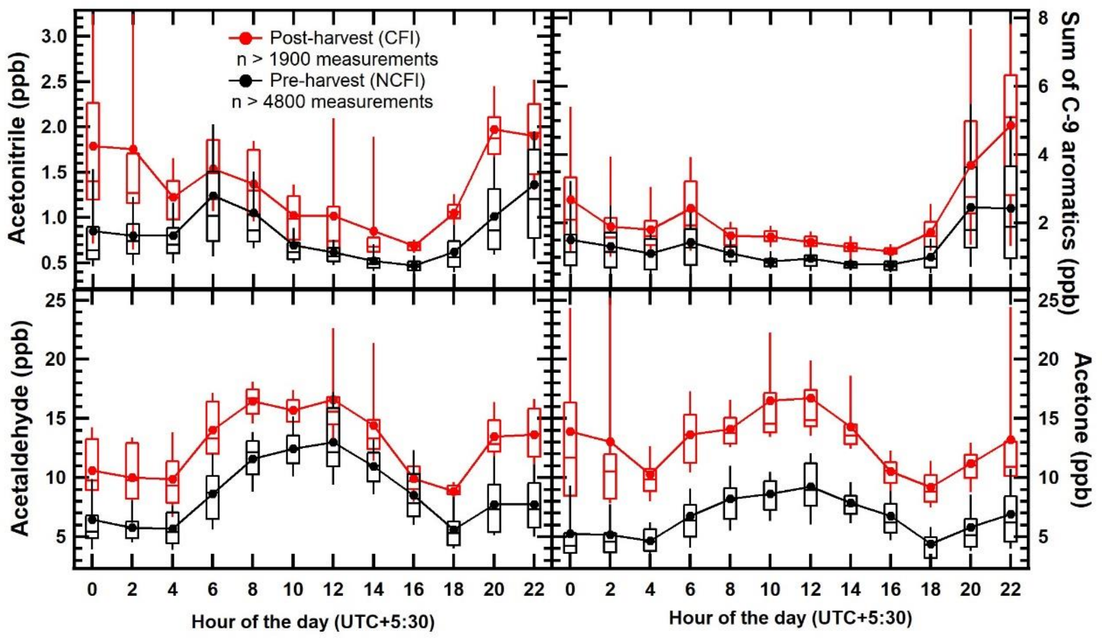

Supplementary Figure 4:

Diel box (25th-75th percentiles) and whisker (10th, 90th percentiles) plots of measured concentrations (in ppb) of m/z 46 (mainly formamide in daytime) and m/z 60 (mainly acetamide in daytime) during the extended pre-harvest NCFI (28 February 2013 to 30 April 2013) and extended post-harvest CFI period (1 May 2013 to 31 May 2013).

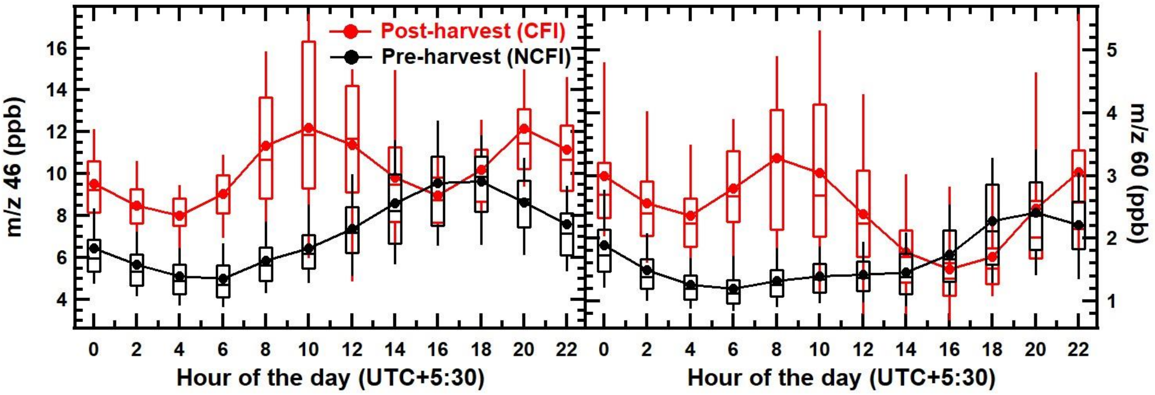

Supplementary Figure 5:

Accuracy of total OH reactivity measurements tested using propane standards of known reactivity during the NCFI and CFI measurement periods. The horizontal error bars represent the uncertainty in the OH reactivity due to the introduced standards (11.2%) and the vertical error bars represent the total uncertainty (18.8 %) of the measured OH reactivity.

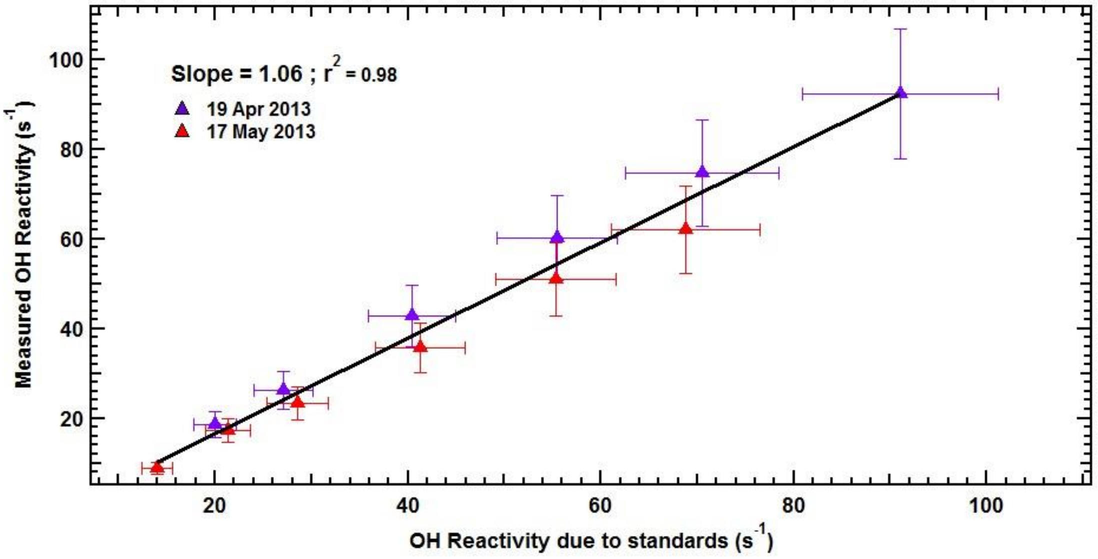

Supplementary Figure 6:

Diel box (25th-75th percentiles) and whiskers (10th, 90th percentiles) profiles of the measured mass concentrations of $PM_{2.5}$ (top panel) and PM_{10} (bottom panel) in the CFI (red) and NCFI (black) periods summer 2013[2].

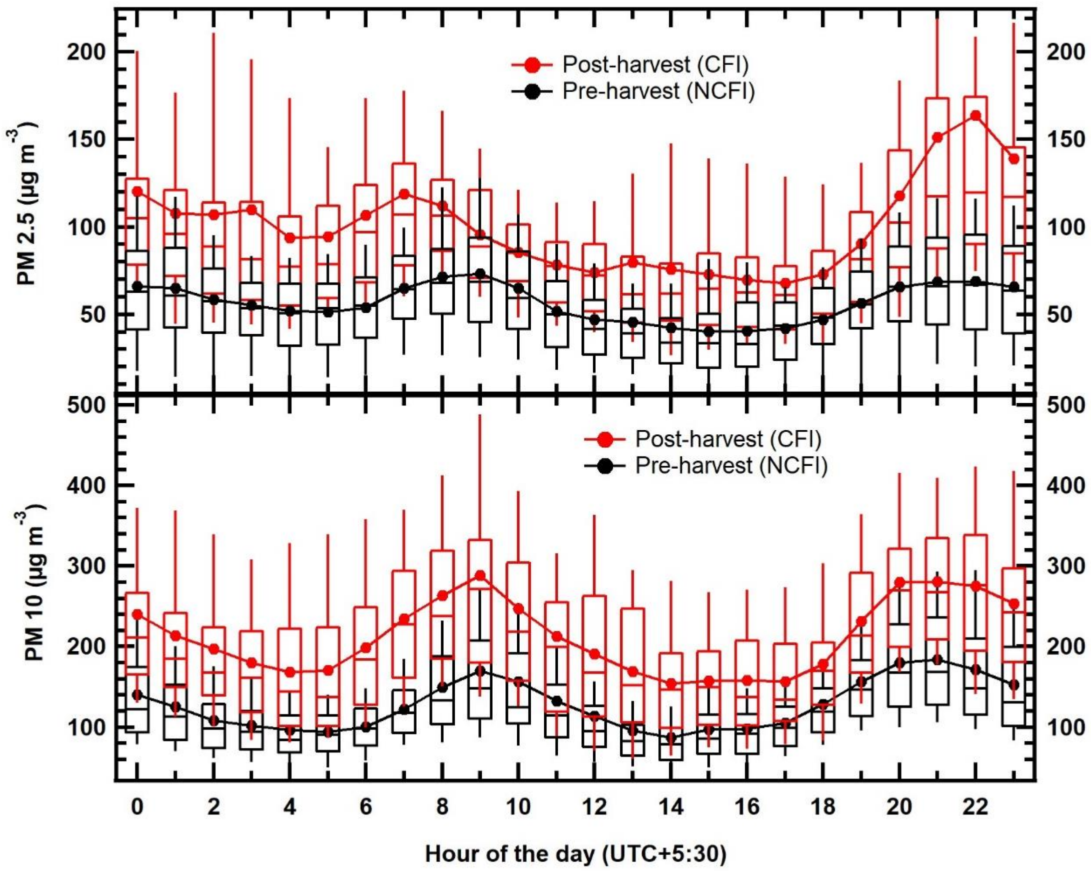

Supplementary Table 1:

Average concentration ± 1σ ambient variability; (75th – 90th percentile) and enhancement ratios of the VOCs and trace gases (in ppb) directly measured along with the total OH reactivity during the pre-harvest non crop-residue fire influenced (NCFI) and post-harvest crop-residue fire influenced (CFI) periods.

Species	Pre-harvest (NCFI) Average ± 1σ (75th – 90th percentile)	Post-harvest (CFI) Average ± 1σ (75th – 90th percentile)	Enhancement ratio
Acetone✓	6.6 ± 1.6 (8.2 – 10.1)	13.1 ± 2.5 (14.5 – 18.2)	2.0
Methyl ethyl ketone✓	1.4 ± 0.4 (1.8 – 2.1)	2.7 ± 0.6 (3.1 – 3.5)	1.9
Sum of C-9 aromatics✓	1.3 ± 0.6 (1.5 – 2.2)	2.3 ± 1.4 (2.6 – 4.6)	1.8
Acetonitrile×	0.8 ± 0.6 (0.9 – 1.5)	1.4 ± 0.8 (1.7 – 2.1)	1.8
Monoterpenes✓	0.6 ± 0.3 (0.8 – 0.9)	1.0 ± 0.3 (1.2 – 1.4)	1.7
Isoprene✓	1.6 ± 0.7 (1.9 – 2.4)	2.5 ± 0.6 (2.9 – 3.3)	1.6
MVK + MACR✓	1.3 ± 0.4 (1.3 – 0.5)	2.0 ± 0.4 (2.4 – 2.7)	1.5
Benzene✓	1.4 ± 1.1 (1.7 – 2.7)	2.1 ± 1.3 (2.8 – 3.8)	1.5
Acetaldehyde✓	8.6 ± 3.4 (11.3 – 13.2)	12.8 ± 3.6 (15.3 – 17.0)	1.5
Sum of C-8 aromatics✓	2.1 ± 1.2 (2.2 – 3.3)	2.6 ± 1.6 (3.0 – 4.8)	1.2
Toluene✓	2.6 ± 2.1 (3.3 – 4.5)	3.2 ± 2.2 (4.1 – 5.7)	1.2
Methanol✓	42.6 ± 8.3 (48.8 – 62.4)	49.9 ± 10.0 (59.8 – 70.7)	1.2
NO_x✓	8.5 ± 7.5 (10.7 – 16.5)	14.4 ± 9.6 (18.7 – 27.1)	1.7
CO✓	325.7 ± 223.0 (370 – 530)	394.8 ± 190.0 (510 – 770)	1.2

A non-parametric Mann–Whitney U test was performed separately for daytime and nighttime hours to check the confidence interval at which the measurement in the two periods represent dataset with different median. The measured VOCs and NO_x pass this test at a confidence interval > 99.9%, whereas for CO the confidence interval for distinct median is 99.9% for daytime and 97.0% for nighttime data. ✓ Species included in the box model. × Species not present in the box model.

Supplementary Table 2:

Average concentration ± 1σ ambient variability; (75th – 90th percentile) and enhancement ratios of the rarely measured VOCs (in ppb) for the extended NCFI and extended CFI periods of summer 2013.

Species	Pre-harvest (NCFI) Average ± 1σ (75th – 90th percentile)	Post-harvest (CFI) Average ± 1σ (75th – 90th percentile)	Enhancement ratio
Nitromethane[×]	0.5 ± 0.1 (0.6 – 0.8)	1.1 ± 0.2 (1.3 – 1.5)	2.2
Hydroxyacetone[✓]	1.6 ± 0.8 (2.0 – 2.5)	3.4 ± 1.6 (4.2 – 5.8)	2.1
Acetic acid[✓]	13.0 ± 7.3 (16.8 – 21.2)	27.4 ± 12.3 (36.0 – 42.9)	2.1
Butane-2,3-dione[✓]	1.2 ± 0.6 (1.6 – 2.0)	2.3 ± 1.1 (2.9 – 3.9)	1.9
2-Furaldehyde/ 2,5-Dimethylfuran[×]	0.7 ± 0.3 (0.8 – 1.1)	1.2 ± 0.6 (1.6 – 2.2)	1.7
Acetamide/ Trimethylamine[×]	1.8 ± 0.7 (2.0 – 2.6)	3.0 ± 1.4 (2.6 – 4.0)	1.7
Ethanol/ Formic acid[✓]	14.5 ± 4.8 (17.1 – 20.5)	24.1 ± 9.1 (29.4 – 35.8)	1.7
Acrolein/ 1-Butene[✓]	3.9 ± 2.3 (4.4 – 6.2)	5.9 ± 3.1 (6.8 – 8.5)	1.5
Formamide/Dimethy lamine/Ethylamine[×]	7.1 ± 2.6 (8.6 – 10.5))	10.2 ± 3.1 (11.6 – 14.8)	1.4
Styrene[✓]	0.3 ± 0.3 (0.3 – 0.4)	0.4 ± 0.5 (0.4 – 0.5)	1.3
Isocyanic acid[×]	1.7 ± 0.6 (2.2 – 2.6)	2.2 ± 0.7 (2.7 – 3.3)	1.3

The measured concentrations in the extended NCFI and extended CFI periods belong to datasets having different medians at a confidence interval greater than 99.9% for the above mentioned VOCs separately for daytime and nighttime hours. [✓] Species included in the box model. [×] Species not present in the box model.

Supplementary Table 3:

Concentrations of C2-C5 alkanes and C2-C4 alkenes for the pre-harvest NCFI and post-harvest CFI periods calculated using their prescribed[3] emission ratio to CO for crop residue fires and the average measured CO concentration in the respective periods.

Hydrocarbon	Pre-harvest (NCFI)	Post-harvest (CFI)
Ethane✓	2.43	3.89
Propane✓	0.89	1.42
i-Butane✓	0.02	0.03
n-Butane✓	0.08	0.12
n-Pentane✓	0.03	0.04
Ethene✓	3.76	6.01
Propene✓	1.79	2.86
1-Butene✓	0.17	0.28
i-Butene✓	0.11	0.17
cis-2-Butene✓	0.07	0.11
trans-2-Butene✓	0.05	0.09
Ethyne✓	1.04	1.66

✓ Species included in the box model.

Supplementary Table 4:

Summary of commonly used box models in atmospheric chemistry investigations listing the chemical mechanisms implemented within the models

Box models	Used chemical mechanisms	Whether amines are included
F0AM/UWCM[4]	MCM (No of gas phase species 5832, No. of gas phase reactions 17224)	No

	Carbon Bond 05 (No. of gas phase species 53, No. of gas phase reactions 156)	
	Carbon Bond 06 (No. of gas phase species 77, No. of gas phase reactions 216)	
	RACM2 (No. of gas phase species 124, No. of gas phase reactions 363)	
	GEOS-CHEM (No. of species 171, No. of reactions 505)	
CAABA[5]	MECCA (No. of gas phase species 286, No. of gas phase reactions 826), MCM, MIME	No
BOXMOX[6]	MCM, MOZART (No. of gas phase species 85, No. of gas phase reactions 157	No
DSMACC[7]	MCM, GEOS-CHEM	No
SAPRC chemical mechanism modeling system (airshed model)[8]	SAPRC-11 (No. of gas phase species 139, No. of gas phase reactions 339)	Yes (limited to reactions of alkylamines with OH radicals. Subsequent chemistry of first generation products (e.g. amides) is not included)

Supplementary Note 1: Sensitivity of box model to choice of rate constants of lumped species

In our model setup we have assumed sum of C-8 aromatics, sum of C-9 aromatics and sum of monoterpenes as separate lumped species with rate constants for reaction with OH radicals equal to that of p-xylene, 1,2,4-trimethylbenzene and alpha pinene, respectively which are the most abundant contributors. We have performed sensitivity runs assuming minimum (case 1) and maximum (case 2) rate constants for the lumped species to provide upper and lower

bounds of the missing OH reactivity. Among the C-8 aromatics included in the MCM 3.3.1, ethylbenzene has the minimum rate constant of reaction with OH radicals (7.0 × 10^{-12} $cm^3molecule^{-1}s^{-1}$ at 298 K) and m-xylene has the maximum rate constant for reaction with OH radicals (2.3 × 10^{-11} $cm^3molecule^{-1}s^{-1}$ at 298 K). Similarly, among the C-9 aromatics included in the MCM 3.3.1, n-propylbenzene has the minimum rate constant of reaction with OH radicals (6.0 × 10^{-12} $cm^3molecule^{-1}s^{-1}$ at 298 K) and 1,3,5-trimethylbenzene has the maximum rate constant for reaction with OH radicals (5.6 × 10^{-11} $cm^3molecule^{-1}s^{-1}$ at 298 K). Among the monoterpenes included in the MCM 3.3.1, alpha pinene has the minimum rate constant of reaction with OH radicals (5.2 × 10^{-11} $cm^3molecule^{-1}s^{-1}$ at 298 K) and limonene has the maximum rate constant for reaction with OH radicals (16.4 × 10^{-11} $cm^3molecule^{-1}s^{-1}$ at 298 K).

In all cases for both NCFI and CFI periods, the change in values of missing OH reactivity relative to present values reported in Fig 3 (5 % missing in NCFI and 39 % missing in CFI), still resulted in no missing OH reactivity in NCFI period (within uncertainty of measurements) and less than 6 % reduction in missing OH reactivity for the CFI period. Thus, the conclusion concerning a large fraction of unexplained OH reactivity in the CFI period remained unaffected.

Supplementary Note 2: Mechanism of atmospheric oxidation of alkylamines to amides, isocyanic acid, nitromethane, dimethylnitramine and dimethylnitrosamine

Mechanism of O_3 initiated atmospheric oxidation of trimethylamine and dimethylamine to nitromethane[9]

$$(CH_3)_3N \xrightarrow[-O_2]{O_3} [(CH_3)_3N^+O^-]^* \xrightarrow[-\cdot CH_3]{} (CH_3)_2NO\cdot \qquad \text{R1}$$

$$(CH_3)_2NO\cdot \xrightarrow[-O_2,-\cdot CH_3]{O_3} CH_3NO_2 \quad \text{R2}$$

$$(CH_3)_2NH \xrightarrow[-O_2]{O_3} [(CH_3)_2N(H)^+O^-]^* \longrightarrow [(CH_3)_2NOH]^* \xrightarrow[-O_2,-\cdot OH]{O_3} (CH_3)_2NO\cdot \quad \text{R3}$$

$$(CH_3)_2NO\cdot \xrightarrow[-O_2,-\cdot CH_3]{O_3} CH_3NO_2 \quad \text{R4}$$

Mechanism of OH radical initiated atmospheric oxidation of alkylamines to amides and isocyanic acid[10]

$$CH_3NH_2 + \cdot OH \rightarrow \cdot CH_2NH_2 + H_2O \quad \text{R5}$$
$$\cdot CH_2NH_2 + O_2 + M \rightarrow \cdot OOCH_2NH_2 + M \quad \text{R6}$$
$$\cdot OOCH_2NH_2 + NO \rightarrow \cdot OCH_2 - NH_2 + NO_2 \quad \text{R7}$$
$$\cdot OCH_2 - NH_2 + O_2 \rightarrow HCONH_2 + \cdot HO_2 \quad \text{R8}$$
$$HCONH_2 + \cdot OH \rightarrow \cdot C(O)NH_2 + H_2O \quad \text{R9a}$$
$$HCONH_2 + \cdot OH \rightarrow \cdot HC(O)NH + H_2O \quad \text{R9b}$$
$$\cdot C(O)NH_2 + O_2 \rightarrow HNCO + \cdot HO_2 \quad \text{R10}$$
$$\cdot HC(O)NH + O_2 \rightarrow HNCO + \cdot HO_2 \quad \text{R11}$$

Mechanism of OH radical initiated atmospheric oxidation of dimethylamine to dimethylnitramine and dimethylnitrosamine[9]

$$(CH_3)_2NH + \cdot OH \rightarrow \cdot (CH_3)_2N + H_2O \quad \text{R12}$$
$$\cdot (CH_3)_2N + NO_2 \rightarrow \cdot (CH_3)_2N - NO_2 \quad \text{R13a}$$
$$\cdot (CH_3)_2N + NO \rightarrow \cdot (CH_3)_2N - NO \quad \text{R13b}$$

Supplementary Note 3: Atmospheric oxidation reactions of hydroxyacetone producing methylglyoxal and HO_2 radicals[11]

$$\cdot OH + CH_3C(O)CH_2OH \xrightarrow{91\%\ yield} CH_3C(O)CHOH\cdot + H_2O \qquad \text{R14}$$

$$CH_3C(O)CHOH\cdot + O_2 \longrightarrow CH_3C(O)CHO + HO_2\cdot \qquad \text{R15}$$

Supporting References

1 Grobe, H., Diepenbroek, M. & Siems, U. PANGAEA, Alfred Wegener Institute, Helmholtz Center for Polar and Marine Research, Bremerhaven, doi: 10.1594/PANGAEA.104840 (2015) (2003).

2 Pawar, H. *et al.* Quantifying the contribution of long-range transport to particulate matter (PM) mass loadings at a suburban site in the north-western Indo-Gangetic Plain (NW-IGP). *Atmos. Chem. Phys.* **15**, 9501-9520, doi:10.5194/acp-15-9501-2015 (2015).

3 Andreae, M. O. & Merlet, P. Emission of trace gases and aerosols from biomass burning. *Global Biogeochem. Cycles* **15**, 955-966, doi:10.1029/2000gb001382 (2001).

4 Wolfe, G. M., Marvin, M. R., Roberts, S. J., Travis, K. R. & Liao, J. The Framework for 0-D Atmospheric Modeling (F0AM) v3.1. *Geosci. Model Dev.* **9**, 3309-3319, doi:10.5194/gmd-9-3309-2016 (2016).

5 Sander, R. *et al.* The atmospheric chemistry box model CAABA/MECCA-3.0. *Geosci. Model Dev.* **4**, 373-380, doi:10.5194/gmd-4-373-2011 (2011).

6 Knote, C. *et al.* Influence of the choice of gas-phase mechanism on predictions of key gaseous pollutants during the AQMEII phase-2 intercomparison. *Atmospheric Environment* **115**, 553-568, doi:https://doi.org/10.1016/j.atmosenv.2014.11.066 (2015).

7 Emmerson, K. M. & Evans, M. J. Comparison of tropospheric gas-phase chemistry schemes for use within global models. *Atmos. Chem. Phys.* **9**, 1831-1845, doi:10.5194/acp-9-1831-2009 (2009).

8 Carter, W. P. L. & Heo, G. Development of revised SAPRC aromatics mechanisms. *Atmospheric Environment* **77**, 404-414, doi:https://doi.org/10.1016/j.atmosenv.2013.05.021 (2013).

9 Nielsen, C. J. *et al.* Atmospheric Degradation of Amines (ADA). Summary report: Photo-oxidation of methylamine, dimethylamine and trimethylamine. CLIMIT project no. 201604. (NILU, Kjeller, 2011).

10 Roberts, J. M. *et al.* New insights into atmospheric sources and sinks of isocyanic acid, HNCO, from recent urban and regional observations. *J. Geophys. Res.* **119**, 1060-1072, doi:10.1002/2013JD019931 (2014).

11 Butkovskaya, N. I., Pouvesle, N., Kukui, A., Mu, Y. & Le Bras, G. Mechanism of the OH-Initiated Oxidation of Hydroxyacetone over the Temperature Range 236−298 K. *The Journal of Physical Chemistry A* **110**, 6833-6843, doi:10.1021/jp056345r (2006).
